# Berberine Attenuates Cell Motility *via* Inhibiting Inflammation-Mediated Lysyl Hydroxylase-2 and Glycolysis

**DOI:** 10.3389/fphar.2022.856777

**Published:** 2022-04-26

**Authors:** Yishan Du, Muhammad Khan, Nana Fang, Fang Ma, Hongzhi Du, Zhenya Tan, Hua Wang, Shi Yin, Xiaohui Wei

**Affiliations:** ^1^ Department of Geriatrics, Affiliated Provincial Hospital of Anhui Medical University, Anhui Medical University, Hefei, China; ^2^ Department of Oncology, the First Affiliated Hospital of Anhui Medical University, Hefei, China; ^3^ Department of Pathophysiology, School of Basic Medical Sciences, Anhui Medical University, Hefei, China; ^4^ Center for Scientific Research of Anhui Medical University, Hefei, China; ^5^ School of Pharmacy, Hubei University of Chinese Medicine, Wuhan, China; ^6^ Anhui Provincial Key Laboratory of Tumor Immunotherapy and Nutrition Therapy, Hefei, China

**Keywords:** berberine, IL-6, LH2, motility, inflammation

## Abstract

Lysyl hydroxylase-2 (LH2) involves in the hydroxylation of telopeptide lysine residues during collagen deposition. Recent studies indicate that interleukin (IL)-6 generated by the chronic inflammation disease may trigger the LH2 expression to accelerate cell motility. Berberine is the alkaloid derived from the traditional Chinese medicine Coptis chinensis, which displays potential anti-inflammatory activity in multiple diseases. The anti-inflammatory activity of berberine has been confirmed by reducing proinflammatory cytokines such as IL-6, IL-8, and IFN-γ. However, whether and how berberine inhibits cellular motility against metastatic spread in triple-negative breast cancer (TNBC) has not been demonstrated, and the underlying mechanism remains unclear. We investigated the effects of berberine on the inflammatory cytokine secretion, cell proliferation, and migration *in vitro* and further explored the effect of berberine on growth and metastasis *in vivo*. Berberine restrained TNBC cell proliferation, motility, and glycolysis process in a dose-dependent way. The secretion of IL-6 was abrogated by berberine in TNBC cells, and IL-6-stimulated cell migration was inhibited by berberine. Mechanistically, berberine remarkably suppressed LH2 expression at both mRNA and protein levels. LH2 depletion led to decreasing the antimotility effect of berberine, and this phenomenon was related to the suppressed glycolysis after LH2 inhibition. Conversely, ectopic restoration of LH2 could further increase the antimotility effect of berberine. Moreover, berberine was confirmed to inhibit cell growth and motility *in vivo*, and the expression of LH2 and glycolytic enzymes was also blocked by berberine *in vivo*. Collectively, this study indicated that berberine could be a promising therapeutic drug *via* regulating LH2 for TNBC.

## Introduction

Metastasis is a complicated disease that occurs due to the interaction between malignant cells and the microenvironment (Waks and Winer, 2019; Li et al., 2021). Inflammation-related changes play particularly vital roles in facilitating the development of metastatic diseases. Despite recent progress, the mechanisms governing metastasis remain incompletely elucidated and the treatment for metastatic diseases is still not well defined.

Lysyl hydroxylases are encoded by distinct procollagen-lysine, 2-oxoglutarate 5-dioxygenase (PLOD) genes. LH2 plays roles in the hydroxylation of telopeptide lysine residues during collagen deposition (Du et al., 2017b; Qi and Xu, 2018). Specifically, the high expression of LH2 involves in the malignant progression of breast, lung, hepatocellular, and renal cell cancers (Noda et al., 2012; Kurozumi et al., 2016; He et al., 2018). Actually, LH2 has been extensively considered as a prometastatic regulator in multiple cancers. In non-small-cell lung cancer (NSCLC), LH2 contributes to NSCLC cell metastasis by facilitating migration and remodeling collagen reorganization (Du et al., 2017a). LH2 is a crucial regulator of integrin β1 to promote the metastatic ability of head and neck squamous cell carcinomas (Ueki et al., 2020). Our previous data indicate that IL-6 facilitates LH2 expression in breast cancer, and a high expression of LH2 in breast cancer leads to epithelial–mesenchymal transition (EMT) and poor prognosis (He et al., 2018). In addition to inducing EMT, it has shown that glycolysis/gluconeogenesis key enzymes are the target genes of LH2 in bladder cancer (Miyamoto et al., 2016). In addition, LH2 has been shown to be a promising target for colorectal cancer metastasis *via* regulating glycolysis enzyme hexokinase 2 (Du et al., 2020). These studies highlight that targeting LH2 may be an effective treatment strategy for metastatic diseases.

Berberine is the main alkaloid in Coptis chinensis, which is extensively founded from many plants, such as Berberidaceae, Papaveraceae, and Rutaceae plants (Fan et al., 2019). Several studies have shown that berberine has exerted multiple biological activities and therapeutic effects, including anti-inflammation, antidiabetes, and antioxidation (Zou et al., 2017; Habtemariam, 2020). Berberine has attracted widespread attention, especially for its potential antitumor properties in various cancers (Chu et al., 2021; Lin et al., 2019). Berberine has shown to inhibit the metastatic ability of breast cancer cells *via* suppress Akt/NF-κB and AP-1 signaling (Kim et al., 2008). Berberine also interferes the cancer metabolism *via* abrogating the fatty acids biosynthesis through regulating the biosynthesis and transportation of citrate (Liu J. et al., 2020). Combining the berberine with emodin synergistically inhibit the aerobic glycolysis and cell proliferation in breast cancer (Ponnusamy et al., 2020). However, little is known about the efficacy of berberine on TNBC cell metastasis, and whether LH2 is the antimetastatic target of berberine in TNBC remains unclear.

This study reveals that berberine plays multifunctional roles in TNBC, *via* suppressing proliferation, migration, inflammation, and glycolysis. It also uncovers berberine metabolically reprograms TNBC cells by inhibiting IL-6-mediated LH2 expression. LH2 plays vital roles in the control of glycolytic metabolism genes. Specifically, berberine suppresses the expression of LH2 and glycolytic enzymes that are necessary for glycolysis catabolism and therefore abrogate TNBC metastasis. Finally, it is validated that berberine inhibits TNBC metastasis *via* reducing LH2 and glycolysis *in vivo*.

## Materials and Methods

### Reagents

The chemicals including berberine and paclitaxel were offered by Meilunbio. The purity of berberine was more than 99.8%. 2-Deoxy-D-glucose (2-DG) was purchased from Meck. The apoptosis detection kit (Annexin V-PI Staining) was purchased from Keygen Biotech (Nanjing, China). The Diff-Stain Set was purchased from Nanjing Jiancheng Bioengineering Institute (Nanjing, China).

### Cell Culture

Human TNBC cell lines MDA-MB-231, MDA-MB-468, and BT-549 were obtained from the Cell Bank of the Institute of Biochemistry and Cell Biology, Chinese Academy of Sciences (Shanghai, China). The cells were maintained in D/F12 medium (Thermo Fisher Scientific, Waltham, MA, United States) containing 10% fetal bovine serum (PAN, Germany), penicillin (50 U/ml), and streptomycin (50 U/ml) at 37°C in a 5% CO_2_ atmosphere.

### Cell Viability Assay

MDA-MB-231, MDA-MB-468, and BT-549 cells were plated at 2000–3000 cells per well into 96-wells plates with a different dose of treated berberine. The cells were added into 20 µL MTT (0.5 mg/ml) and incubated for 4 h. The culture medium was removed and following that 150 μL DMSO was added and mixed. Using a spectrophotometer (PE, Enspire), the absorbance at 490 nm was detected.

### Apoptosis Detection

The cells were treated with berberine for 48 h. Cell apoptosis was further evaluated by Annexin V-FITC and PI staining with flow cytometry.

### Lactic Acid Production

The TNBC cells were treated with 1.25, 2.5, and 5 μM berberine for 48 h, and then, the cell culture media were replaced with a fresh medium and cultured for 24 h. The media were collected and measured according to manufacturer’s instructions of the Lactic Acid Production Detection Kit (KeyGen, Nanjing, China). The assay result was tested by using a spectrophotometer (PE, Enspire) at 530 nm.

### Glucose Uptake Assay

The TNBC cells were treated with 1.25, 2.5, and 5 μM berberine for 48 h, and the culture medium was collected to measure the glucose uptake. The glucose uptake assay was further detected by using the Glucose Mensuration Reagent Kit (KeyGen, Nanjing, China) according to the manufacturer’s instruction. The assay results were performed by using a spectrophotometer at 505 nm.

### Measurement of Cellular ATP

The TNBC cells at the density of 2000 cells/well were seeded into 96-wells plates and exposed with berberine for 48h. The ATP concentration was measured by using CellTiter-Glo® Kit (Promega).

### Respiration Assays

Oxygen consumption rate (OCR) and extracellular acidification rate (ECAR) of the TNBC cells were measured by the Seahorse XF24 Extracellular Flux Analyzer (Agilent) after treating with berberine. In brief, the TNBC cells were seeded into XF 24-well microplate wells at a density of 15,000 cells/well and incubated overnight until the cells become completely adherent in a CO_2_-free incubator. Prior to assay, the TNBC cells were washed with XF basal medium containing 2 mM glucose, 4 mM pyruvate, and 3 mM glutamine at pH7.4. The TNBC cells were supplemented in XF basal medium and incubated in a incubator for 1 h. 15 min prior the plate loading, the TNBC cells were added 10 μM oligomycin, 20 μM FCCP, and 5 μM rotenone to quantify OCR and ECAR. OCR and ECAR values were normalized to the protein content.

### Western Blotting and Real-Time PCR

The total protein of the TNBC cells was extracted *via* RIPA lysis buffer containing protease inhibitors and phosphatase inhibitors. The total protein was separated *via* 8–15% SDS-PAGE and transferred onto a PVDF membrane (Millipore). Then, the total protein was incubated with 5% BSA for 1 h at room temperature to block the PVDF membranes. The primary antibodies were incubated overnight at 4°C, and then, the PVDF membranes were incubated with secondary antibody for 1 h at room temperature. The antigen–antibody complexes were detected by an enhanced chemiluminescence (ECL).

TRIzol reagent (Vazyme, Nanjing, China) was used to isolate the total cellular RNA. Reverse transcription of RNA into cDNA was performed using the HiScript QRT SuperMix for qPCR Kit (Vazyme, Nanjing, China), and the cDNA was amplified using an RT-PCR amplification kit (Vazyme, Nanjing, China). The primers were designed using Primer Premier 5 software (primer sequences are shown in Table 1). The 2^−ΔΔCt^ method was used to perform the analysis with β-actin as the reference gene.

**TABLE 1:** The primers for q-PCR.

Gene name	Forward sequence	Reverse sequence
Human		
LH2	GAC​AGC​GTT​CTC​TTC​GTC​CTC​A	CTC​CAG​CCT​TTT​CGT​GGT​GAC​T
β-Actin	CAC​CAT​TGG​CAA​TGA​GCG​GTT​C	AGG​TCT​TTG​CGG​ATG​TCC​ACG​T
LDHA	GGA​TCT​CCA​ACA​TGG​CAG​CCT​T	AGA​CGG​CTT​TCT​CCC​TCT​TGC​T
ALDOA1	GAC​ACT​CTA​CCA​GAA​GGC​GGA​T	GGT​GGT​AGT​CTC​GCC​ATT​TGT​C
PGK1	CCG​CTT​TCA​TGT​GGA​GGA​AGA​AG	CTC​TGT​GAG​CAG​TGC​CAA​AAG​C
PKM2	ATG​GCT​GAC​ACA​TTC​CTG​GAG​C	CCT​TCA​ACG​TCT​CCA​CTG​ATC​G
PFKFB	TAC​CGA​CCT​CTT​GAC​CCA​GAC​A	TAA​ATG​GTG​CGA​GGC​TGG​ACG​T
PGAM	GCT​CTG​CCC​TTC​TGG​AAT​GAA​G	ATA​CCA​GTC​GGC​AGG​TTC​AGC​T

### RNA Interference

Lipofectamine 3000 (Thermo Fisher Scientific) was performed for cell transfection. The LH2 siRNA and control group were purchased from Hanbio Biotechnology company (Shanghai). The sequences of siLH2#1 and siLH2#2 are 5′- GGA​ACA​CUA​UGC​UGA​UCA​ATT -3′ and 5′- GCA​GUA​GAU​GUC​CAU​CCA​ATT -3′, respectively. Sequence of NC is 5′-UUC​UCC​GAA​CGU​GUC​ACG​UTT-3′.

### Orthotopic Mammary Fat Pad Breast Tumor Model

The female BALB/c mice (5–6 weeks) were purchased from the Model Animal Research Center of Nanjing University. All the animal procedures were approved by the Experimental Animal Care Commission of Anhui Medical University (Approval number: LLSC20200016). The mice were anesthetized intraperitoneally with isoflurane inhalation, then 4T1 mouse triple-negative breast cancer cells (5 × 10^5^ cells per mouse) were orthotopically injected into the inguinal mammary fat pad. One week later, the mice were randomly divided into groups according to the tumor volume (five or six mice per group). The mice were intra-gastrically (ig) administered with berberine (7.5, 15 and 30 mg/kg, 6 times a week) in 0.5% CMC-Na solution or with paclitaxel which was used as a positive drug (20 mg/kg) twice a week *via* the tail-vein injection. The tumor volume was calculated by the formula V = (L × W^2^)/2. After 21 days, the mice were sacrificed and the lungs were fixed by formalin and embedded for hematoxylin and eosin staining. Metastatic foci were quantified after the H&E staining of five mice per group and are presented as the mean ±SD. The immunohistochemical staining was measured as described below.

### Immunohistochemistry Assay

4 μm sections of the paraffin-embedded tumor tissue were used for immunohistochemistry (IHC). LH2 (dilution 1:200, Proteintech), PGK1 (dilution 1:200, Cell Signaling Technologies), and LDHA (Cell Signaling Technologies, dilution at 1:150) were used as the primary antibodies, and the sections were visualized with DAB and hematoxylin and eosin (H&E) staining.

### Statistical Analysis

The GraphPad Prism 8.0 software was used to calculate the statistical significance. One-way ANOVA was used for the comparison of multiple groups. *p*-value of <0.05 was considered statistically significant. The results in the present study were expressed as the mean ± SD for the triplicate experiments.

## Results

### Berberine Inhibits Proliferation and Migration, but Fails to Induce Cell Apoptosis in TNBC Cells.

**FIGURE 1:**
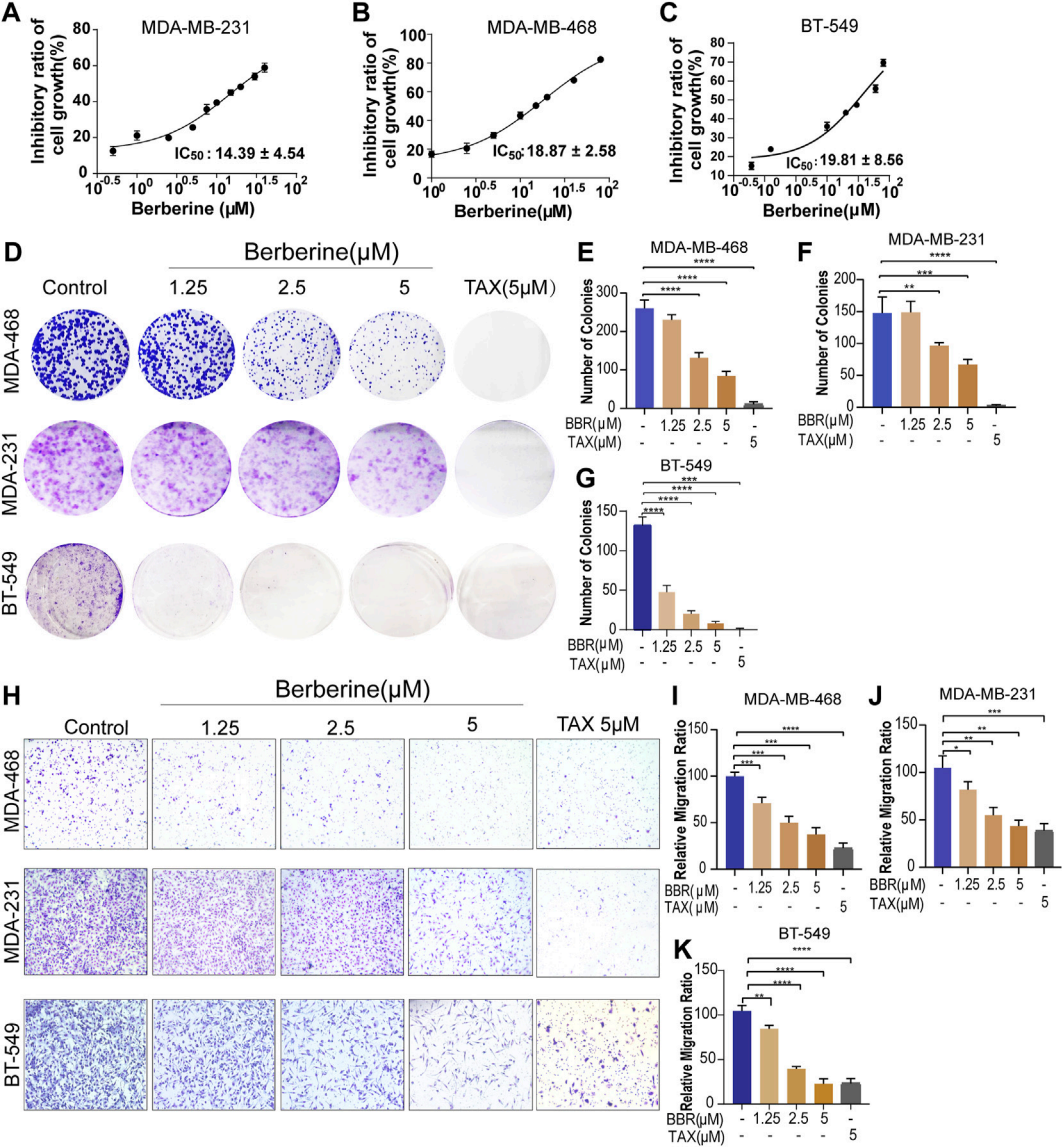
Berberine inhibits the proliferation and migration of TNBC cells. **(A–C)** Viability of the TNBC cells treated with berberine for 72 h was analyzed by the MTT assay. The cell viability data were normalized by comparing to control cells, and GraphPad Prism eight Software was used to analyze the data. **(D)** Antigrowth effect of berberine on the TNBC cells was determined by colony formation. The TNBC cells were treated with berberine or TAX for 3 days, then removed the drugs and cultured the cells in a drug-free medium for 6–8 days. Finally, crystal violet was used to stain the colonies. **(E–G)** The percentage of colonies was calculated by GraphPad Prism eight Software. TAX (5 μM) was used as the positive control. **(H–K)** Representative images and quantification of migration of the berberine-treated TNBC cells. TAX was used as a positive control drug. 10 × scale bars, 200 μm. Error bars were means ± SD. *p* < 0.05, *p* < 0.01, *p* < 0.0001 vs. control.

To determine the effect of berberine on TNBC cells growth, the TNBC cells were treated with different concentration of berberine and the MTT assay was used. The results of cell viability showed the 50% inhibitory concentration (IC_50_) of berberine on MDA-MB-231, MDA-MB-468, and BT-549 were 14.39 ± 4.54 µM, 18.87 ± 2.58 µM, and 19.81 ± 8.56 µM, respectively (Figures 1A–C). Therefore, we chose the concentration of berberine less than the IC_50_ to examine the inhibition effect on proliferation, apoptosis, and migration.

The colony formation assay was used to explore the antiproliferation effect of berberine in the TNBC cells. The results showed that berberine could significantly reduce the number of cell colonies (Figures 1D–G). Then, the effect of berberine on cell apoptosis was detected by the flow cytometry. It was showed that berberine barely induced apoptosis of the TNBC cells as compared with the control group (Supplementary Figures S1A,B).

We further investigated whether berberine prevented the migratory ability of the TNBC cells, the transwell migration assays indicated that berberine could suppress the migratory ability of MDA-MB-231, MDA-MB-468, and BT-549 cells in a dose-dependent manner (Figures 1H–K). Collectively, these results suggested that berberine could inhibit cell proliferation and migration, rather than inducing cell apoptosis in the TNBC cells.

### Berberine Inhibits Aerobic Glycolysis in TNBC Cells

**FIGURE 2:**
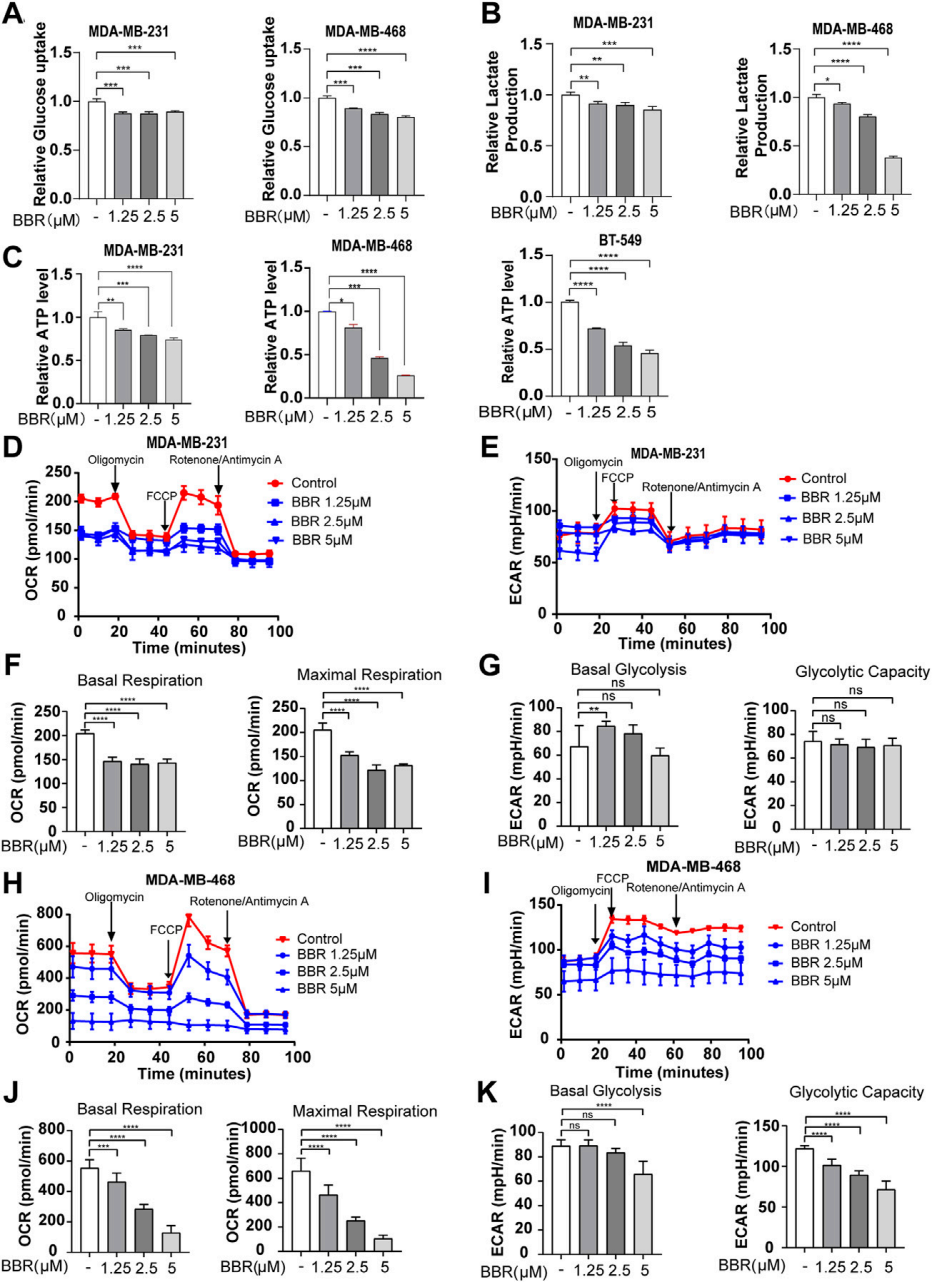
Berberine dampens glycolysis in the TNBC cells. **(A–C)**. Glucose uptake, lactate secretion, and ATP generation level in the TNBC cells after the treatment with berberine (1.25, 2.5, and 5 μM) for 48 h **(D–E,H–I)**. Effects of berberine on the oxygen consumption ratio (OCR) and extracellular acid ratio (ECAR) in MDA-MB-231 and MDA-MB-468 cells. The TNBC cells were treated with berberine for 48 h, then the cells were collected for the OCR and ECAR as determined by the Seahorse XF24. **(F,J)**. The basal respiration and maximal respiration capacity of the TNBC cells with berberine treatment were analyzed by using GraphPad Prism 8 software. **(G,K)** The basal glycolysis and glycolytic capacity of the TNBC cells with berberine treatment were analyzed by GraphPad Prism 8 software. The data are presented as the mean ± S.D. of at least three independent measurements. **p < 0.01, ***p < 0.001, ****p < 0.0001 vs. the control group.

The abovementioned results showed that berberine could suppress the malignant phenotype of the TNBC cells. Metabolic reprogramming is considered as the hallmark of cancer, and TNBC has displayed the glycolytic phenotype with elevated glucose uptake. Since berberine has been reported to exert roles on regulating the glucose metabolism, we speculated that the antimigratory effect occurred by berberine might through the regulating glycolytic metabolism—the significant metabolic pattern of the TNBC cells. The glucose content assay showed that berberine treatment led to reducing glucose consumption in the TNBC cells (Figure 2A). Subsequently, lactate, the final metabolite product of glucose in the glycolysis process, and cellular ATP levels also dramatically reduced in the berberine-treated TNBC cells (Figures 2B,C).

**FIGURE 3:**
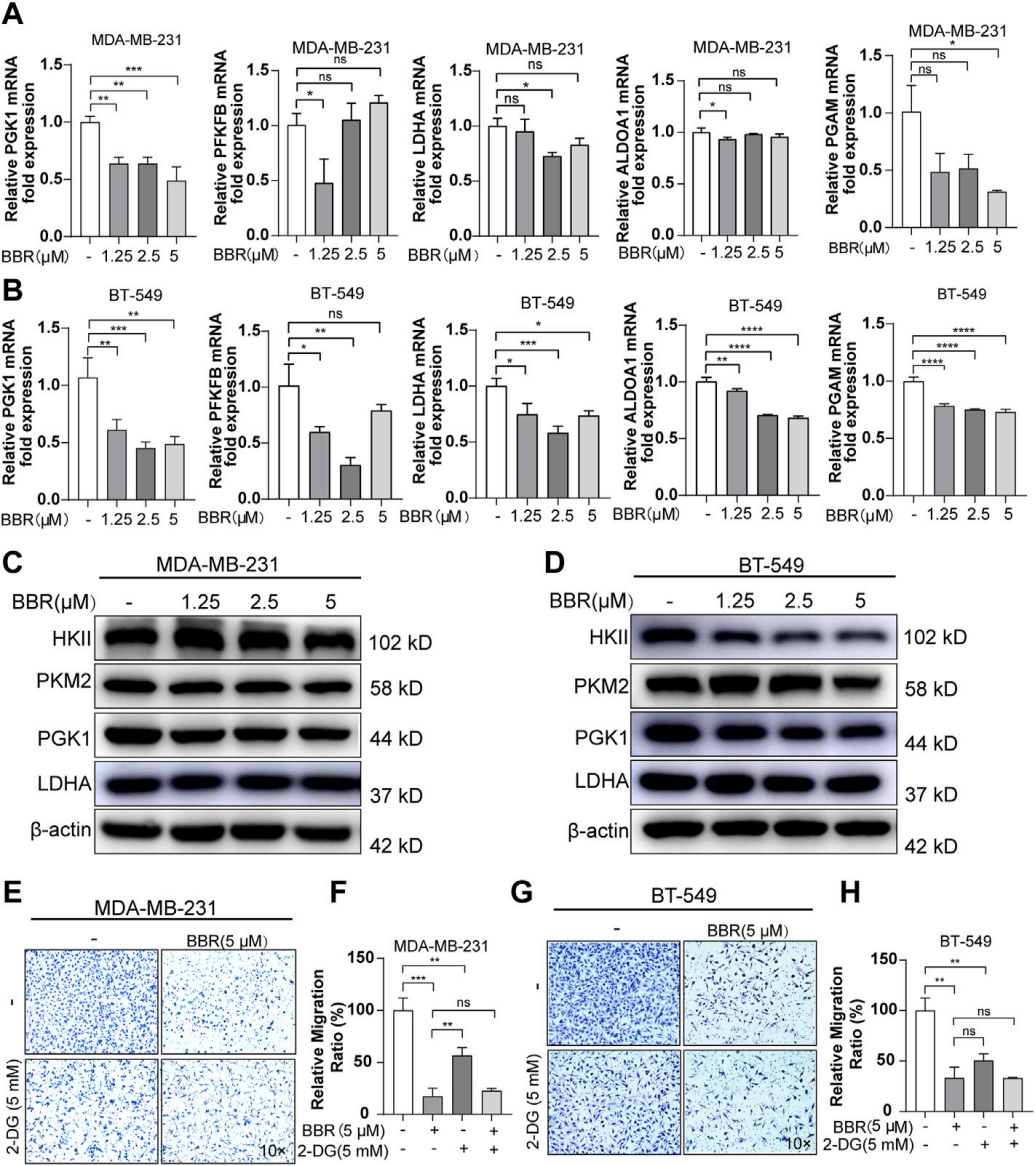
Berberine suppresses aerobic glycolysis thus inhibiting the migration of TNBC cells. **(A–B)** The mRNA levels of PGK1, LDHA, PFKFB, ALDOA1, and PGAM in the TNBC cells after berberine treatment for 48 h. The data were normalized to β-actin expression. **(C–D)**. Protein levels of HK2, PKM2, PGK1, and LDHA in the TNBC cells after berberine (1.25, 2.5, 5 μM) treatment for 48 h β-actin was used as a loading control. **(E–H)** Representative images of MDA-MB-231 or BT-549 cells were pretreated with or without 2-DG (5 mM) for 6 h, then the cells were treated with berberine (5 μM) for another 48 h, following which the cells were collected for the migration assay. 10 × scale bars, 200 μm. The data are presented as the mean ± SD of three independent measurements. *p < 0.05, **p < 0.01, ***p < 0.001 vs. control.

To further demonstrate the role of berberine on the glucose metabolic process, we measured the oxygen consumption rate (OCR) and extracellular acidification rate (ECAR) using the XF24 Extracellular Flux Analyzer. The results indicated that berberine suppressed the mitochondrial oxygen consumption and glycolytic capability of the TNBC cells (Figures 2D–G, 2H-K). Moreover, the gene expression of key enzymes involved in the glycolysis process including PGK1, PGAM, PFKFB, ALDOA1, and LDHA were inhibited after berberine treatment (Figures 3A,B). Consistently, HKII, PGK1, and PKM2 protein expressions were decreased after the treatment with berberine (Figures 3C,D).

2-Deoxy-D-glucose (2-DG) is a glucose analogue, which can interfere with the glycolysis metabolism. To assess the role of glycolysis on mediating the antimigratory effect by berberine in the TNBC cells, using 2-DG to suppress the glucose metabolism in MDA-MB-231 and BT-549 cells was followed by the migration assays. 2-DG could block the migration of MDA-MB-231 and BT-549 cells, while pretreating with 2-DG attenuated the antimetastasis effect of berberine. Actually, as compared with the berberine treatment, the antimigratory ratio of berberine decreased with 2-DG pretreatment both in MDA-MB-231 (from 83 to 34%) and BT-549 cells (from 67 to 17%) (Figures 3E–H). Although, the remaining antimetastasis effect of berberine may mediate *via* the other signaling pathways aside from the inhibition of glycolysis. Together, these results indicated that berberine could partially exert antimigration effect *via* inhibiting the glycolysis process in the TNBC cells.

### Berberine Suppresses TNBC Cell Migration *via* Inhibiting IL-6 Secretion and LH2 Expression

**FIGURE 4:**
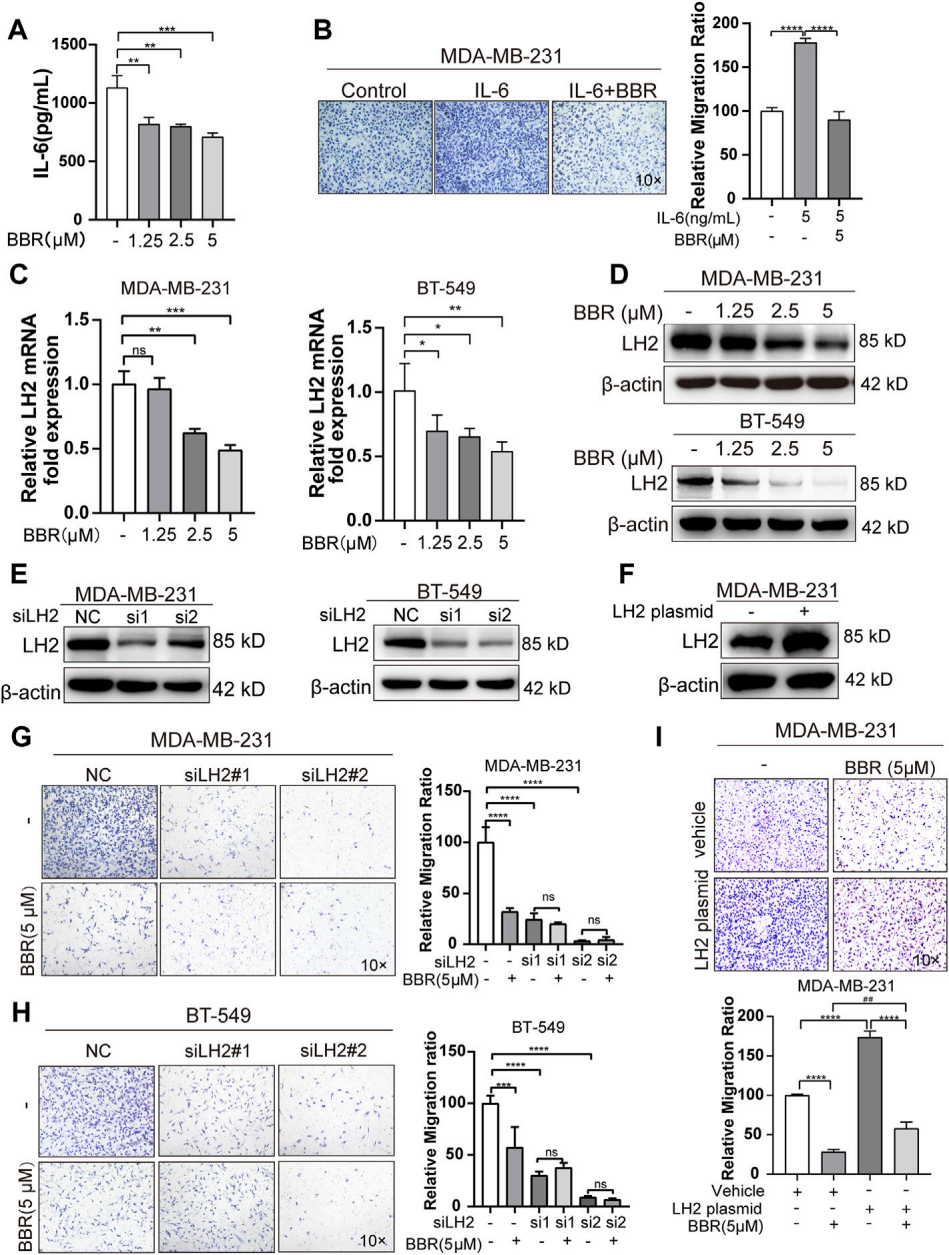
Berberine inhibits the migration of TNBC cells in a LH2-dependent manner. **(A–B)**. The secretion of IL-6 was detected by using the IL-6 Elisa kit. MDA-MB-231 cells were cultured with or without berberine (1.25, 2.5 and 5 μM) for 48 h, then the supernatant medium was collected for the detection of IL-6 levels. **(B)** Recombination human IL-6 protein (5 ng/ml) was used to stimulate MDA-MB-231 cells for 6 h, and berberine (5 μM) was used to treat MDA-MB-231 cells for 48 h, following which the cells were collected for transwell assay for 12 h. **(C–D)** LH2 mRNA and protein expression in the MDA-MB-231 and BT-549 cells in the presence of berberine (1.25, 2.5, 5 μM) were measured by q-RT-PCR and Western blot analysis. **(E)** Silencing of LH2 and validated the knockdown efficiency at protein levels. **(F)** Overexpression of LH2 and validated the efficiency at protein levels. **(G–H)** Representative images of the MDA-MB-231 or BT-549 cells treated with berberine (5 μM) or not after LH2 silencing. **(I)** Representative images of the MDA-MB-231cells treated with berberine (5 μM) or not after the LH2 overexpression. 10 × Scale bars, 200 μm. All results were representative of three independent experiments. **p < 0.01, ***p < 0.001 vs. control, nc, vehicle cells or LH2 overexpression cells. ###*p* < 0.001 vs. vehicle cells treated with berberine.

The TNBC cells have been proved to aberrant expression of LH2, which leads to the metastasis of TNBC cells. Our previous study indicated that IL-6-mediated the high expression of LH2 in the TNBC cells. As berberine is a well-known anti-inflammation drug, we try to explore the role of berberine on IL-6 secretion by the Elisa analysis. The results showed that berberine could suppress the secretion of IL-6 in the TNBC cells (Figure 4A). Furthermore, we found that the recombinant human IL-6-stimulated TNBC cells migration was inhibited by berberine (Figure 4B), suggesting that berberine could abrogate IL-6 signaling in the TNBC cells.

Given that IL-6 has been shown to modulate the LH2 expression in the TNBC cells to support metastasis, we determined whether the antimigratory effect of berberine was mediated by LH2. We assessed the LH2 expression in the TNBC cells after the berberine treatment for 48 h. The results demonstrated that LH2 was decreased at the transcription and translation levels by treating with berberine (Figures 4C– D). In order to confirm that LH2 inhibition by berberine indeed causes the antimigratory efficiency of the TNBC cells. By silencing and overexpressing LH2 in the TNBC cells, we further verified the LH2 expression in the TNBC cells (Figures 4E,F). It was showed that knocking down of LH2 could remarkably inhibit the TNBC cells migration, but berberine failed to increase the antimigratory ability of the TNBC cells with LH2 silencing (Figures 4G,H). Inversely, the migration ability of the TNBC cells was enhanced after the ectopic LH2 expression (1.7 folds) (Figure 4G). Consistently, the antimigratory role of berberine was also increased in the LH2-overexpressed TNBC cells compared with the vehicle cells treated with berberine (from 71.6 to 130%) (Figure 4I). Together, these results suggested that berberine exerted the antimigratory effect *via* inhibiting the IL-6 secretion and LH2 expression in the TNBC cells.

### Inhibition of LH2 Constrains TNBC Cell Migration Primarily Through Glycolysis Modulation

The previous studies indicated that the activity of LH2 was associated with the glycolytic metabolism in the colorectal and bladder cancer (Miyamoto et al., 2016; Du et al., 2020). The results abovementioned showed that the inhibition of glycolysis by berberine could suppress the aggressive phenotype of the TNBC cells. These findings suggested that LH2 inhibition by berberine may lead to decreasing glycolytic abilities as well as suppressing the malignant phenotype of the TNBC cells.

**FIGURE 5:**
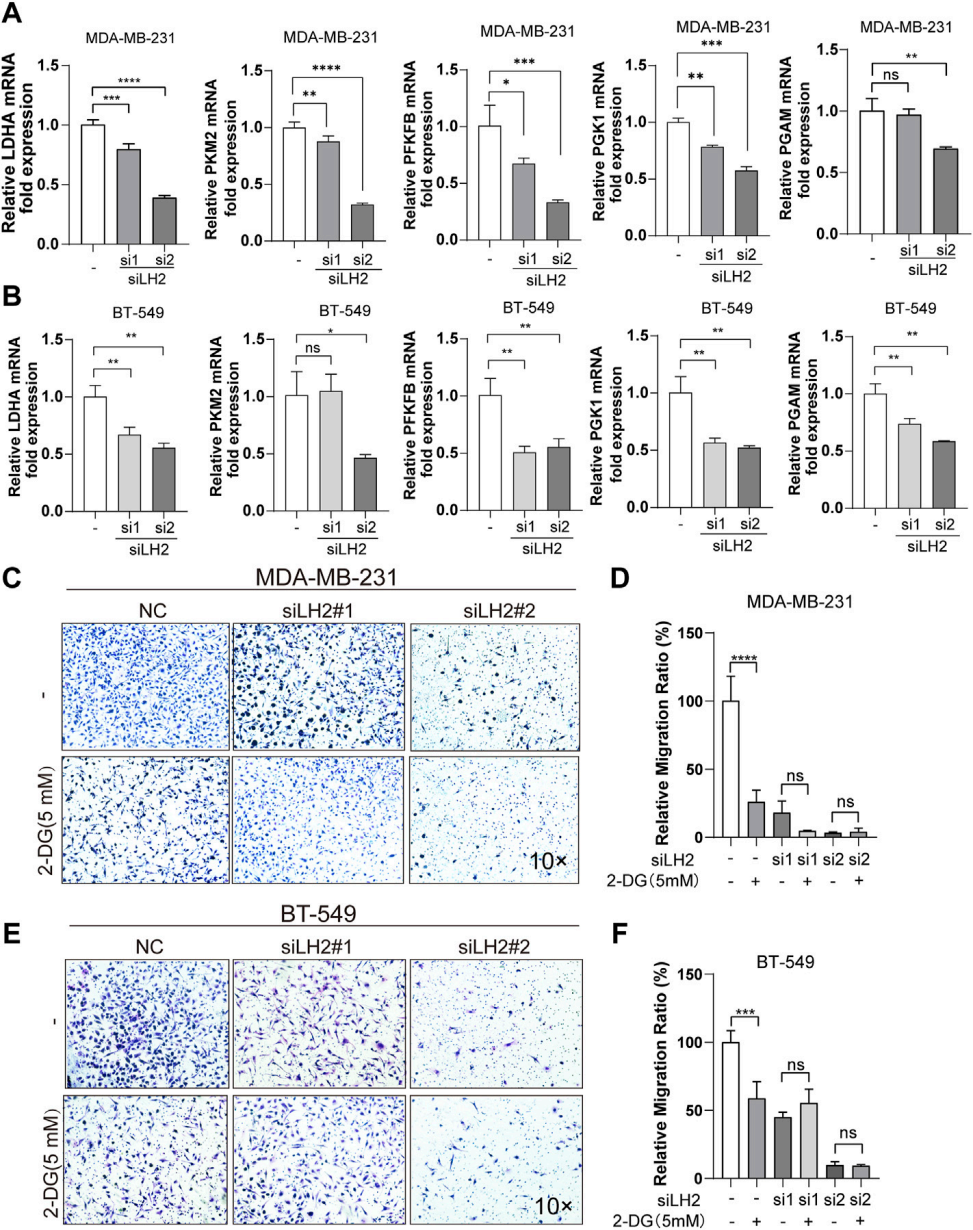
Inhibition of the LH2 constrains TNBC cell migration primarily through glycolysis modulation. **(A–B)** mRNA expression of glycolytic enzymes including PGK1, LDHA, PFKFB, PKM2, and PGAM in the MDA-MB-231 or BT-549 cells after LH2 silencing. **(C–F)** Representative images of the MDA-MB-231 or BT-549 cells treated with 2-DG (5 mM) or not after LH2 silencing. All the results were representative of three independent experiments. 10 × scale bars, 200 μm **p < 0.01, ***p < 0.001, ****p < 0.0001 vs. the control or nc cells.

To further illustrate the role of LH2 on modulating glycolysis in the TNBC cells, we explored the key enzymes that participated in the glycolytic process after the knockdown of LH2 in the TNBC cells. The gene expression of the glycolytic enzymes including PGK1, LDHA, PKM2, and PGAM were dramatically decreased after the LH2 knockdown (Figures 5A–B). Subsequently, to further confirm the glycolysis involved in the promigratory effect of LH2, 2-DG was used to inhibit glycolysis in the LH2 knockdown cells, and the results showed that 2-DG inhibited the migration of the negative control cells, but 2-DG failed to increase the antimigratory ability of the LH2 silencing cells (Figures 5C–F). Together, these results imply that the inhibition of LH2 could constrain the TNBC migration *via* blocking the glycolysis process.

### Berberine Suppresses LH2 Expression and Inhibits Metastasis in the Orthotopic Mammary Fat Breast Tumor Model

**FIGURE 6:**
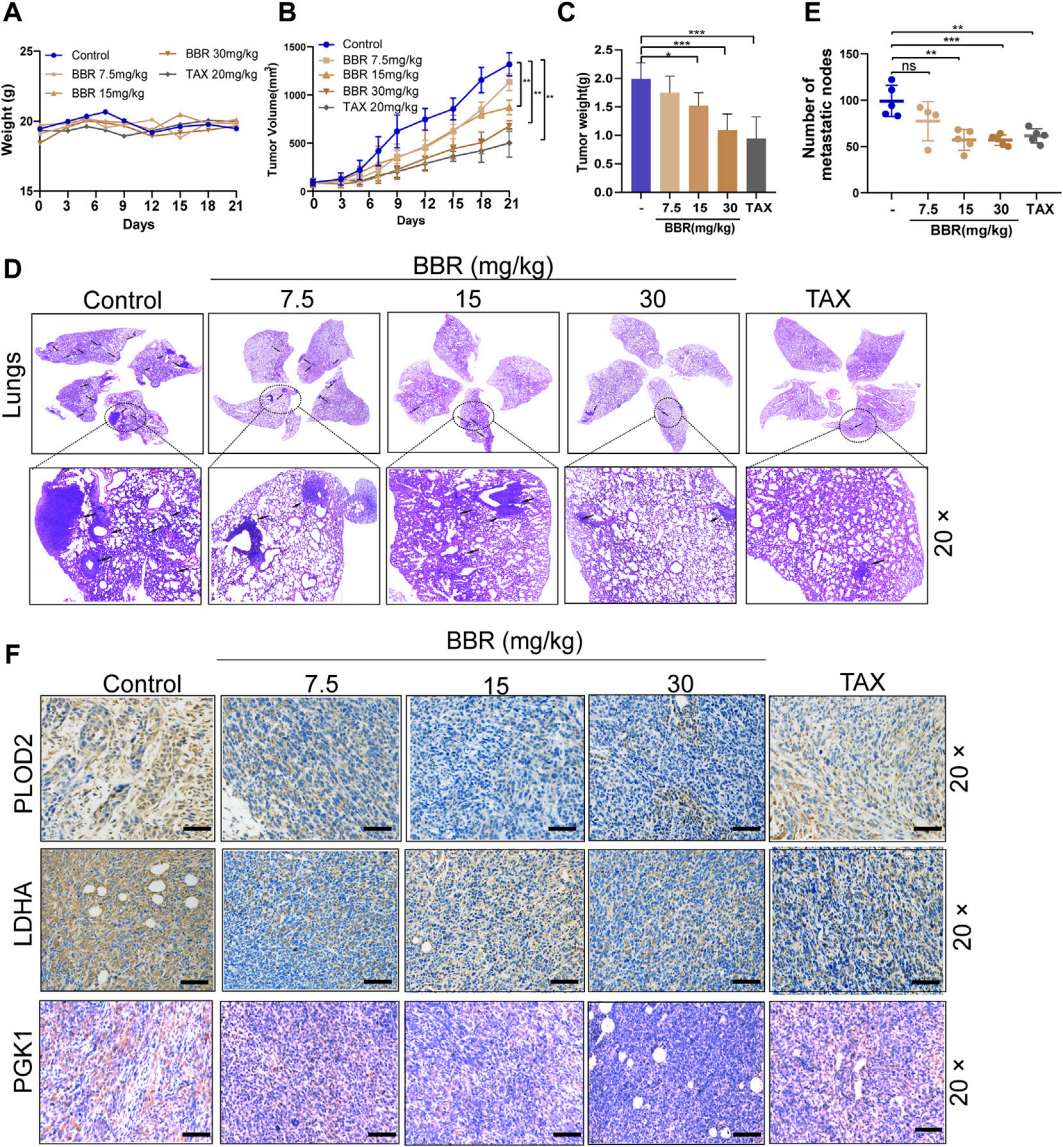
Berberine attenuates TNBC growth and inhibits the metastasis in the orthotopic mammary fat breast tumor model. **(A)** The alteration of body weights in different treatment groups. Error bars were the means ± SD, *n* = 5 or 6. **(B–C)** Tumor volume curves of tumor growth and tumor weight after orthotopic injection of the 4T1 cells. Berberine (15 mg/kg, 30 mg/kg) administration inhibited the growth of 4T1 breast tumors, and TAX (20 mg/kg) was used as the positive control. Error bars were the mean ± SD. *n* = 5 or 6 per group, *p* < 0.01. **(D)** Representative pictures of the lungs were detected by H&E staining. Arrows indicate metastatic colonization. **(E)** Quantification of metastatic foci in lung was calculated, *n* = 5 or 6 per group. *p* < 0.01, *p* < 0.001 vs. the control. **(F)** The LH2, LDHA, and PGK1 expression in tumors were detected by the immunohistochemistry staining, respectively. 20 × scale bars, 100 μm.

We established the breast cancer orthotopic implantation model to investigate the antitumor effect of berberine by using the mouse breast cancer 4T1 cells. Compared with the control group, the body weight slightly fluctuated after the treatment with berberine but without a statistical significance (Figure 6A). It was shown that the tumor volume and weight were dose-dependently decreased *in vivo* following the treatment with berberine (15 and 30 mg/kg) or TAX (20 mg/kg) than the control group (Figures 6B,C). We further assessed the effect of berberine on the tumor metastasis ability by the staining of lung metastases sections. It was showed that berberine could reduce the number of lung metastatic foci at 15 mg/kg and 30 mg/kg (Figures 6D,E). Simultaneously, berberine decreased the expression of LH2 in the orthotopic tumor tissues (Figure 6F). In addition, immunohistochemistry of the tumor sections showed that the expression levels of key glycolytic enzymes PGK1 and LDHA decreased *via* the treatment with berberine (Figure 6F). Collectively, these results revealed that berberine inhibited triple-negative breast cancer metastasis *via* the downregulating LH2 and glycolytic enzymes expression, which was consistent with the *in vitro* results.

## Discussion

TNBC is an invasive type of breast cancer which is still lacking effective therapies, and there is an urgent need to find reliable treatment (Barzaman et al., 2020; Liu Y. et al., 2020). In the present study, it is shown that berberine prevents proliferation and metastasis of the TNBC cells. Mechanistically, we reveal that berberine abrogates the IL-6 secretion and LH2 expression in the TNBC cells, which further mediates the antimetastasis effect. In addition, our results indicate that the inhibition of LH2 expression leads to abrogating glycolysis metabolism in the TNBC cells, inhibiting the LH2-mediated glycolysis responsible for the antimigratory role of berberine.

The previous studies have shown that berberine and its derivatives exert antitumor effects in cancer (Liu Y. et al., 2020; Xia et al., 2021). The effect of berberine on inhibiting proliferation and stimulating caspase-dependent apoptosis of the tumor cells mediates varieties of signaling pathways, such as MAPK and AMPK (Refaat et al., 2015; Zhang et al., 2019). Berberine is also shown to inhibit mTOR, AKT, and MAPK pathways, thereby inducing autophagy (Zhang et al., 2020). However, in this study, we showed that berberine exerted no effect on the TNBC cells apoptosis, which may be because of the low dosage of berberine used in this study. Berberine has demonstrated to inhibit the breast cancer cells migration by inhibiting IL-8 transcription in an EGFR-dependent way (Kim et al., 2018). Similarly, our present study showed that the antimigratory efficiency of berberine on the TNBC cells increased in a dose-dependent manner.

In the present study, our results showed that berberine could decrease the expression of LH2 both at the mRNA and protein levels. However, as the crystal structure of LH2 has not been identified completely, it is still not sure whether berberine combined with LH2 directly or indirectly regulates the LH2 expression. Interestingly, berberine inhibited the secretion of IL-6 by the TNBC cells, at least, this study confirms that berberine could regulate LH2 indirectly by regulating the IL-6 secretion. Several studies have suggested that abnormal expression of LH2 promote multiple types of tumor metastasis (Du et al., 2017b; Yang et al., 2020). LH2 is a critical regulator to stabilize integrin by the LH2-mediated hydroxylation, enabling integrin β1 to initiate tumor metastasis (Ueki et al., 2020). Our previous study indicated that LH2 was highly expressed in the TNBC cells than the non-TNBC cells, which further led to cancer metastasis by facilitating EMT (He et al., 2018). Interestingly, LH2 is a novel regulator to promote the aerobic glycolysis and tumor progression in the colorectal cancer by upregulating hexokinase 2 (Du et al., 2020), as LH2 is regulated by HIF-1α, which may mediate the aerobic glycolysis. The present study showed that silencing LH2 suppressed glycolytic enzymes including PGK1, LDHA, and PKM2, and abrogating of glycolysis failed to increase the antimigratory effect of the LH2 silencing cells. These results suggest that LH2 exerted antitumor effect *via* regulating the glycolysis metabolism. Furthermore, LH2 is the key enzyme to hydroxylate lysine in the telopeptides of procollagens and remodel collagen fibers. However, the present study has not evaluated the role of berberine on the TNBC cells collagen organization or formation. We will try to explore whether berberine plays roles in the collagen deposition and organization in the future study.

Cancer progression is related to glucose metabolism reprogramming, as metabolic alternations of the TNBC cells lead to metastasis competence (Sun et al., 2020; Varghese et al., 2020). It was shown that berberine inhibits glucose consumption, ATP production, and lactate production in the TNBC cells. In addition, the oxygen consumption rate and extracellular acidification rate in the TNBC cells were suppressed by berberine, suggesting that berberine might inhibit glycolysis process and mitochondria metabolism in the TNBC cells. Further study showed that berberine inhibited several glycolytic enzymes, especially PGK1 was suppressed both at the mRNA and protein levels by berberine. It has shown that PGK1-mediated tumor progression through regulating the glucose metabolism, as a vital enzyme to generate ATP in the glycolytic pathway (Fu and Yu, 2020). The inhibition of PGK1 could significantly reverse the epithelial-mesenchymal transformation process in the breast cancer (Li et al., 2018). In our research, we confirmed that berberine inhibits the LH2 expression which result in the abrogation of glycolytic process, thereby suppressing the TNBC cells metastasis. However, the specific mechanism for LH2 regulated glycolysis in the TNBC cells was not explored clearly in this study. Previous studies have shown that LH2 could activate the STAT3 signaling to upregulate the HK2 expression in the colorectal cancer cells, thus mediating glycolysis (Du et al., 2020). The present study showed that LH2 inhibition led to multiple glycolytic enzymes suppression rather than HK2, suggesting that LH2 may regulate specific regulators that control the glycolysis process. We will further explore the specific mechanism of LH2 for regulating glycolysis in the TNBC cells.

## Conclusion

In summary, this study suggested that berberine could inhibit the glycolysis process *via* suppressing the IL-6-mediated LH2 expression, which further inhibits the TNBC metastasis.

## Data Availability

The original contributions presented in the study are included in the article/Supplementary Material, further inquiries can be directed to the corresponding authors.

## Abbreviations

IL-6, interleukin-6; LH2, lysyl hydroxylase-2; PGK1, phosphoglycerate kinase one; PGAM, phosphoglycerate mutase kinase; PKM2, pyruvate kinase isozyme type M; LDHA, lactate dehydrogenase A.

## References

[B1] Barzaman K. Karami J. Zarei Z. Hosseinzadeh A. Kazemi M. H. Moradi-Kalbolandi S. (2020). Breast Cancer: Biology, Biomarkers, and Treatments. Int. Immunopharmacol 84, 106535. 10.1016/j.intimp.2020.106535 32361569

[B2] Chu Z. Huo N. Zhu X. Liu H. Cong R. Ma L. (2021). FOXO3A-induced LINC00926 Suppresses Breast Tumor Growth and Metastasis through Inhibition of PGK1-Mediated Warburg Effect. Mol. Ther. 29, 2737–2753. 10.1016/j.ymthe.2021.04.036 33940159 PMC8417517

[B3] Du H. Chen Y. Hou X. Huang Y. Wei X. Yu X. (2017a). PLOD2 Regulated by Transcription Factor FOXA1 Promotes Metastasis in NSCLC. Cell Death Dis 8, e3143. 10.1038/cddis.2017.553 29072684 PMC5680920

[B4] Du H. Pang M. Hou X. Yuan S. Sun L. (2017b). PLOD2 in Cancer Research. Biomed. Pharmacother. 90, 670–676. 10.1016/j.biopha.2017.04.023 28415047

[B5] Du W. Liu N. Zhang Y. Liu X. Yang Y. Chen W. (2020). PLOD2 Promotes Aerobic Glycolysis and Cell Progression in Colorectal Cancer by Upregulating HK2. Biochem. Cel Biol 98, 386–395. 10.1139/bcb-2019-0256 31742425

[B6] Fan J. Zhang K. Jin Y. Li B. Gao S. Zhu J. (2019). Pharmacological Effects of Berberine on Mood Disorders. J. Cel Mol Med 23, 21–28. 10.1111/jcmm.13930 PMC6307759 30450823

[B7] Fu Q. Yu Z. (2020). Phosphoglycerate Kinase 1 (PGK1) in Cancer: A Promising Target for Diagnosis and Therapy. Life Sci. 256, 117863. 10.1016/j.lfs.2020.117863 32479953

[B8] Habtemariam S. (2020). Berberine Pharmacology and the Gut Microbiota: A Hidden Therapeutic Link. Pharmacol. Res. 155, 104722. 10.1016/j.phrs.2020.104722 32105754

[B9] He J. Y. Wei X. H. Li S. J. Liu Y. Hu H. L. Li Z. Z. (2018). Adipocyte-derived IL-6 and Leptin Promote Breast Cancer Metastasis via Upregulation of Lysyl Hydroxylase-2 Expression. Cell Commun Signal 16, 100. 10.1186/s12964-018-0309-z 30563531 PMC6299564

[B10] Kim S. Choi J. H. Kim J. B. Nam S. J. Yang J. H. Kim J. H. (2008). Berberine Suppresses TNF-Alpha-Induced MMP-9 and Cell Invasion through Inhibition of AP-1 Activity in MDA-MB-231 Human Breast Cancer Cells. Molecules 13, 2975–2985. 10.3390/molecules13122975 19052522 PMC6244848

[B11] Kim S. You D. Jeong Y. Yu J. Kim S. W. Nam S. J. (2018). Berberine Down-Regulates IL-8 Expression through Inhibition of the EGFR/MEK/ERK Pathway in Triple-Negative Breast Cancer Cells. Phytomedicine 50, 43–49. 10.1016/j.phymed.2018.08.004 30466991

[B12] Kurozumi A. Kato M. Goto Y. Matsushita R. Nishikawa R. Okato A. (2016). Regulation of the Collagen Cross-Linking Enzymes LOXL2 and PLOD2 by Tumor-Suppressive microRNA-26a/b in Renal Cell Carcinoma. Int. J. Oncol. 48, 1837–1846. 10.3892/ijo.2016.3440 26983694 PMC4809659

[B13] Li C. I. Zhang Y. Cieślik M. Wu Y. M. Xiao L. Cobain E. (2021). Cancer Cell Intrinsic and Immunologic Phenotypes Determine Clinical Outcomes in Basal-like Breast Cancer. Clin. Cancer Res. 27, 3079–3093. 10.1158/1078-0432.CCR-20-3890 33753452

[B14] Li L. Liang Y. Kang L. Liu Y. Gao S. Chen S. (2018). Transcriptional Regulation of the Warburg Effect in Cancer by SIX1. Cancer Cell 33, 368–e7. 10.1016/.j.ccell.2018.01.01010 29455928

[B15] Lin Y. S. Chiu Y. C. Tsai Y. H. Tsai Y. F. Wang J. Y. Tseng L. M. (2019). Different Mechanisms Involved in the Berberine-Induced Antiproliferation Effects in Triple-Negative Breast Cancer Cell Lines. J. Cel Biochem 120, 13531–13544. 10.1002/jcb.28628 30957305

[B16] Liu J. Luo X. Guo R. Jing W. Lu H. (2020a). Cell Metabolomics Reveals Berberine-Inhibited Pancreatic Cancer Cell Viability and Metastasis by Regulating Citrate Metabolism. J. Proteome Res. 19, 3825–3836. 10.1021/acs.jproteome.0c00394 32692565

[B17] Liu Y. Hua W. Li Y. Xian X. Zhao Z. Liu C. (2020b). Berberine Suppresses colon Cancer Cell Proliferation by Inhibiting the SCAP/SREBP-1 Signaling Pathway-Mediated Lipogenesis. Biochem. Pharmacol. 174, 113776. 10.1016/j.bcp.2019.113776 31874145

[B18] Miyamoto K. Seki N. Matsushita R. Yonemori M. Yoshino H. Nakagawa M. (2016). Tumour-suppressive miRNA-26a-5p and miR-26b-5p Inhibit Cell Aggressiveness by Regulating PLOD2 in Bladder Cancer. Br. J. Cancer 115, 354–363. 10.1038/bjc.2016.179 27310702 PMC4973152

[B19] Noda T. Yamamoto H. Takemasa I. Yamada D. Uemura M. Wada H. (2012). PLOD2 Induced under Hypoxia Is a Novel Prognostic Factor for Hepatocellular Carcinoma after Curative Resection. Liver Int. 32, 110–118. 10.1111/j.1478-3231.2011.02619.x 22098155

[B20] Ponnusamy L. Kothandan G. Manoharan R. (2020). Berberine and Emodin Abrogates Breast Cancer Growth and Facilitates Apoptosis through Inactivation of SIK3-Induced mTOR and Akt Signaling Pathway. Biochim. Biophys. Acta Mol. Basis Dis. 1866, 165897. 10.1016/j.bbadis.2020.165897 32682817

[B21] Qi Y. Xu R. (2018). Roles of PLODs in Collagen Synthesis and Cancer Progression. Front Cel Dev Biol 6, 66. 10.3389/fcell.2018.00066 PMC6031748 30003082

[B22] Refaat A. Abdelhamed S. Saiki I. Sakurai H. (2015). Inhibition of P38 Mitogen-Activated Protein Kinase Potentiates the Apoptotic Effect of Berberine/tumor Necrosis Factor-Related Apoptosis-Inducing Ligand Combination Therapy. Oncol. Lett. 10, 1907–1911. 10.3892/ol.2015.3494 26622773 PMC4533710

[B23] Sun X. Wang M. Wang M. Yu X. Guo J. Sun T. (2020). Metabolic Reprogramming in Triple-Negative Breast Cancer. Front. Oncol. 10, 428. 10.3389/fonc.2020.00428 32296646 PMC7136496

[B24] Ueki Y. Saito K. Iioka H. Sakamoto I. Kanda Y. Sakaguchi M. (2020). PLOD2 Is Essential to Functional Activation of Integrin β1 for Invasion/Metastasis in Head and Neck Squamous Cell Carcinomas. iScience 23, 100850. 10.1016/j.isci.2020.100850 32058962 PMC6997870

[B25] Varghese E. Samuel S. M. Líšková A. Samec M. Kubatka P. Büsselberg D. (2020). Targeting Glucose Metabolism to Overcome Resistance to Anticancer Chemotherapy in Breast Cancer. Cancers (Basel) 12, 2252. 10.3390/cancers12082252 PMC7464784 32806533

[B26] Waks A. G. Winer E. P. (2019). Breast Cancer Treatment: A Review. JAMA 321, 288–300. 10.1001/jama.2018.19323 30667505

[B27] Xia Y. Chen S. Cui J. Wang Y. Liu X. Shen Y. (2021). Berberine Suppresses Bladder Cancer Cell Proliferation by Inhibiting JAK1-STAT3 Signaling via Upregulation of miR-17-5p. Biochem. Pharmacol. 188, 114575. 10.1016/j.bcp.2021.114575 33887260

[B28] Yang B. Zhao Y. Wang L. Zhao Y. Wei L. Chen D. (2020). Identification of PLOD Family Genes as Novel Prognostic Biomarkers for Hepatocellular Carcinoma. Front. Oncol. 10, 1695. 10.3389/fonc.2020.01695 33014843 PMC7509443

[B29] Zhang C. Sheng J. Li G. Zhao L. Wang Y. Yang W. (2019). Effects of Berberine and its Derivatives on Cancer: A Systems Pharmacology Review. Front. Pharmacol. 10, 1461. 10.3389/fphar.2019.01461 32009943 PMC6974675

[B30] Zhang Q. Wang X. Cao S. Sun Y. He X. Jiang B. (2020). Berberine Represses Human Gastric Cancer Cell Growth *In Vitro* and *In Vivo* by Inducing Cytostatic Autophagy via Inhibition of MAPK/mTOR/p70S6K and Akt Signaling Pathways. Biomed. Pharmacother. 128, 110245. 10.1016/j.biopha.2020.110245 32454290

[B31] Zou K. Li Z. Zhang Y. Zhang H. Y. Li B. Zhu W. L. (2017). Advances in the Study of Berberine and its Derivatives: a Focus on Anti-inflammatory and Anti-tumor Effects in the Digestive System. Acta Pharmacol. Sin 38, 157–167. 10.1038/aps.2016.125 27917872 PMC5309756

