# Supplementary material for: Berberine Attenuates Cell Motility via Inhibiting Inflammation-Mediated Lysyl Hydroxylase-2 and Glycolysis
Source: Front Pharmacol. 2022 Apr 26;13:856777. doi: 10.3389/fphar.2022.856777 (PMC9086160; doi:10.3389/fphar.2022.856777)
Supplement: Supplementary file 1 [file DataSheet1.pdf]

Supplementary figure 1

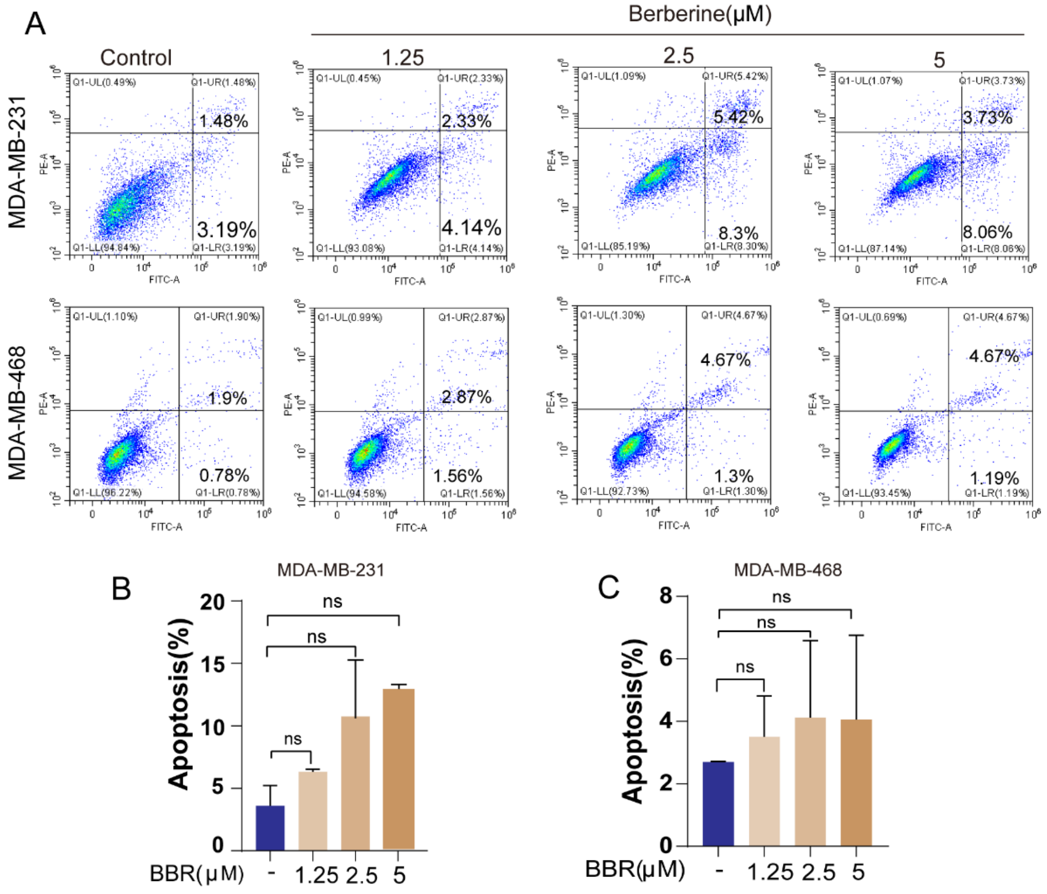

Supplementary figure 1. Berberine fails to induce apoptosis of TNBC cells. (A) Cell apoptosis profiles of MDA-MB-231, MDA-MB-468 cells treated with berberine for 72 h. Annexin V/PI double staining assay was performed by flow cytometry to detect the percentage of apoptotic cells. (B) The percentage of apoptosis was obtained for MDA-MB-231, MDA-MB-468 cells, and data analyses were performed by GraphPad Prism 8 Software.
