# Supplementary material for: Berberine Attenuates Cell Motility via Inhibiting Inflammation-Mediated Lysyl Hydroxylase-2 and Glycolysis
Source: Front Pharmacol. 2022 Apr 26;13:856777. doi: 10.3389/fphar.2022.856777 (PMC9086160; doi:10.3389/fphar.2022.856777)
Supplement: Supplementary file 2 [file DataSheet2.docx]

**Original Data link:**

**Berberine Attenuates Cell Motility via Inhibiting Inflammation-mediated Lysyl Hydroxylase-2 and Glycolysis**

**Figure 1:**

Fig.1A: <https://www.jianguoyun.com/p/DcgJHYcQssmeChjW7KoE>

Fig.1B: <https://www.jianguoyun.com/p/DTtDBFYQssmeChjY7KoE>

Fig.1C: <https://www.jianguoyun.com/p/DZg8DjgQssmeChjb7KoE>

Fig.1D: <https://www.jianguoyun.com/p/DVzRRbUQssmeChjc7KoE>

Fig.1E-G: <https://www.jianguoyun.com/p/DauPuYoQssmeChjd7KoE>

Fig.1H: <https://www.jianguoyun.com/p/DZLZqe8QssmeChje7KoE>

Fig.1I-K: <https://www.jianguoyun.com/p/DQt4ZlcQssmeChj37KoE>

**Figure 2:**

Fig.2A-2B: <https://www.jianguoyun.com/p/DejYxrQQtMmeChjDhawE>

Fig.2C: <https://www.jianguoyun.com/p/DT2yKGsQtMmeChjEhawE>

Fig.2D: <https://www.jianguoyun.com/p/Da0dW4oQtMmeChjFhawE>

Fig.2E: <https://www.jianguoyun.com/p/DUdfgCIQtMmeChjIhawE>

Fig.2F: <https://www.jianguoyun.com/p/DThJWUcQtMmeChjJhawE>

Fig.2G: <https://www.jianguoyun.com/p/DcPbYDsQtMmeChjKhawE>

Fig.2H: <https://www.jianguoyun.com/p/Da8JANIQtMmeChjLhawE>

Fig.2I: <https://www.jianguoyun.com/p/DVmt9KQQtMmeChjMhawE>

Fig.2J: <https://www.jianguoyun.com/p/Df_qfzgQtMmeChjOhawE>

Fig.2K: <https://www.jianguoyun.com/p/DSnJYq8QtMmeChjPhawE>

**Figure 3:**

Fig.3A: <https://www.jianguoyun.com/p/DWihw9EQtsmeChjQhawE>

Fig.3B: <https://www.jianguoyun.com/p/Dfy9wbAQtsmeChjRhawE>

Fig.3C: <https://www.jianguoyun.com/p/DWRw3ScQtsmeChjShawE>

Fig.3D: <https://www.jianguoyun.com/p/DZji6S0QtsmeChjThawE>

Fig.3E: <https://www.jianguoyun.com/p/DQlCO3MQtsmeChjUhawE>

Fig.3F: <https://www.jianguoyun.com/p/DesgBsAQtsmeChjVhawE>

Fig.3G: <https://www.jianguoyun.com/p/DeDi9LsQtsmeChjWhawE>

Fig.3H: <https://www.jianguoyun.com/p/DU1j1EMQtsmeChjXhawE>

**Figure 4:**

Fig.4A: <https://www.jianguoyun.com/p/DS81FScQt8meChiMrasE>

Fig.4B: <https://www.jianguoyun.com/p/Dfm-5TAQt8meChiNrasE>

Fig.4C: <https://www.jianguoyun.com/p/DTBP4zsQt8meChiOrasE>

Fig.4D: <https://www.jianguoyun.com/p/Dadr0bcQt8meChiPrasE>

Fig.4E: <https://www.jianguoyun.com/p/DfGKxioQt8meChiQrasE>

Fig.4F: <https://www.jianguoyun.com/p/DUZliHwQt8meChiRrasE>

Fig.4G: <https://www.jianguoyun.com/p/DfREixgQt8meChiUrasE>

Fig.4H: <https://www.jianguoyun.com/p/DcfuRawQt8meChjsjrEE>

Fig.4I: <https://www.jianguoyun.com/p/DX-fFywQt8meChjwjrEE>

**Figure 5:**

Fig.5A: <https://www.jianguoyun.com/p/DZIwJMUQ1MmeChiZrasE>

Fig.5B: <https://www.jianguoyun.com/p/DSMe6ywQ1MmeChiarasE>

Fig.5C: <https://www.jianguoyun.com/p/DeT2VLMQ1MmeChibrasE>

Fig.5D: <https://www.jianguoyun.com/p/Dczi8XIQ1MmeChicrasE>

Fig.5E: <https://www.jianguoyun.com/p/DTtNLn8Q1MmeChidrasE>

Fig.5F: <https://www.jianguoyun.com/p/DagGsXgQ1MmeChierasE>

**Figure 6:**

Fig.6A-C: <https://www.jianguoyun.com/p/DXJIn3sQuMmeChjehawE>

Fig.6D: <https://www.jianguoyun.com/p/DW0OWncQuMmeChjfhawE>

Fig.6E: <https://www.jianguoyun.com/p/DWnF_6EQuMmeChjghawE>

Fig.6F: <https://www.jianguoyun.com/p/DRN4_ZUQuMmeChjhhawE>
